# Trends, Associations, and Antimicrobial Resistance of *Salmonella* Typhi and Paratyphi in Pakistan

**DOI:** 10.4269/ajtmh.18-0145

**Published:** 2018-07-25

**Authors:** Jai K. Das, Rumina Hasan, Afia Zafar, Imran Ahmed, Aamer Ikram, Summiya Nizamuddin, Saleel Fatima, Nauman Akbar, Faisal Sultan, Zulfiqar A. Bhutta

**Affiliations:** 1 Division of Women and Child Health, The Aga Khan University, Karachi, Pakistan;; 2 Department of Pathology, The Aga Khan University, Karachi, Pakistan;; 3 Armed Forces Institute of Pathology, National University of Medical Sciences, Rawalpindi, Pakistan;; 4 Department of Pathology, Shaukat Khanum Memorial Cancer Hospital and Research Centre, Lahore, Pakistan;; 5 Department of Medicine, Shaukat Khanum Memorial Cancer Hospital and Research Centre, Lahore, Pakistan;; 6 Centre of Excellence in Women and Child Health, The Aga Khan University, Karachi, Pakistan;; 7 Centre for Global Child Health, The Hospital for Sick Children, Toronto, Canada;; 8 Centre for Global Child Health, The Hospital for Sick Children, Toronto, Canada

## Abstract

Typhoid remains a major cause of morbidity and mortality in endemic countries. This review analyzed typhoid burden changes in Pakistan and its association with contextual factors. A retrospective cohort study on blood culture–positive typhoid and antibiotic resistance was conducted from three tertiary hospitals and contextual factor data obtained from primary household surveys. *Salmonella* Typhi/Paratyphi positivity rates were estimated and trend analysis was carried out using positive cases out of total number of blood cultures performed. Contextual factors’ associations were determined through bivariate correlation analysis, using STATA (SataCorp, College Station, TX). We report a total of 17,387 *S.* Typhi*–positive* and 8,286 *S.* Paratyphi A and B–positive specimens from 798,137 blood cultures performed. The results suggest an overall decline in typhoid incidence as *S.* Typhi positivity rates declined from 6.42% in 1992 to 1.32% in 2015 and *S.* Paratyphi (A and B) from 1.29% to 0.39%. Subgroup analysis suggests higher *S.* Typhi prevalence in adults older than 18 years, whereas *S.* Paratyphi is greater in children aged 5–18 years. The relative contribution of *S.* Paratyphi to overall confirmed cases increased from 16.8% in 1992 to 23% in 2015. The analysis suggests high burden of fluoroquinolone resistance and multidrug-resistant *S.* Typhi strains. Statistically significant associations of water, sanitation indicators, and literacy rates were observed with typhoid positivity. Despite some progress, typhoid remains endemic and a strong political will is required for targeted typhoid control strategies. A multipronged approach of improving water, sanitation and hygiene in combination with large-scale immunization in endemic settings of Pakistan could help reduce burden and prevent epidemics.

## INTRODUCTION

Typhoid fever, also known as enteric fever, is caused by *Salmonella enterica* serotype Typhi (*Salmonella* Typhi) and *S. enterica* serotype Paratyphi (*Salmonella* Paratyphi) A, B, and C.^1^ Typhoid is transmitted by the fecal–oral route and characterized by fever accompanied by chills, nausea, diffuse abdominal pain, rash, anorexia, and diarrhea or constipation^2^ and on physical examination, hepatomegaly, splenomegaly, and relative bradycardia are common.^3^

Typhoid remains a major global cause of morbidity and mortality, particularly in low- and middle-income countries.^4^ Typhoid is common in areas that are overcrowded and have poor sanitation. According to the World Health Organization (WHO) report published in 2014, the global annual incidence of typhoid was approximately 21 million cases with approximately 222,000 annual typhoid-related deaths.^5^ A systematic review on the global morbidity and mortality due to typhoid and paratyphoid fever estimated the incidence of typhoid fever as < 0.1/100,000 in Central and Eastern Europe and Central Asia and 724.6/100,000 in sub-Saharan Africa. The incidence of paratyphoid fever was 0.8/100,000 in North Africa and Middle East, 77.4/100,000 in sub-Saharan Africa and South Asia.^6^ In a study focusing on five Asian countries, the annual incidence of typhoid cases (per 100,000 person years) was estimated to be 24.2 in Vietnam, 29.3 in China, 180.3 in Indonesia, and 412.9 and 493.5 in Pakistan and India, respectively.^7^ It was also reported that in the higher burden countries such as Pakistan, India, and Indonesia, the incidence of typhoid fever in children aged 2–5 years was the same as that for children aged 5–15 years.^7^ A literature review of typhoid in developing countries since 1960 reported the frequency of intestinal perforation as a complication of typhoid to be about 3% with a mortality rate of 39.6%.^8^

The strategies which are effective to prevent the spread of typhoid include access to safe drinking water, hygiene, and uncontaminated food supplies,^9^ and strategies to improve microbial quality of drinking water are more effective than improving sanitation.^5^ Vaccines also have the potential to prevent typhoid fever and currently two vaccines are available: the Ty21a vaccine which is a live attenuated oral vaccine and the Vi parenteral vaccine.^10^ Both vaccines have equal efficacy and are given to children aged ≥ 2 years.^11^ Antibiotics are the mainstay for the treatment of typhoid but *S.* Typhi and *S.* Paratyphi are now showing resistance to the traditional antimicrobials.^12^ Third-generation cephalosporins are now commonly used for the treatment of *S.* Typhi and *S.* Paratyphi because antimicrobial resistance (AMR) has developed to a combination of traditional first-line drugs known as multi-drug resistance (resistance to ampicillin, chloramphenicol, and trimethoprim–sulfamethoxazole) and fluoroquinolones. Azithromycin is also used as an alternative to third-generation cephalosporin as it is cost-effective.^10^

The objective of this study is to analyze the observed changes in the burden of typhoid in Pakistan and its association with various contextual factors and to evaluate the trends in AMR. We evaluated the data from three large tertiary care hospitals of Pakistan which have laboratories across various cities of Pakistan. The findings of this study will help guide the development of a research and implementation agenda for the prevention and control of typhoid in Pakistan and countries with similar epidemiology and burden. It will also aid in priority setting for research and implementation to tackle the global burden of typhoid.

## TYPHOID—PAKISTAN CONTEXT

Pakistan has remained highly endemic for typhoid fever over the last several decades. According to a study^13^ conducted in low socioeconomic areas of the city of Karachi from 2002–2003, the annual incidence of typhoid fever cases was estimated between 252 and 503 per 100,000 child years which was four times higher than the WHO-defined criteria,^4^ and the incidence of typhoid was higher in children aged 2–10 years (78%) when compared with children aged 2–5 years (35%).^13^ This was consistent with results from another study conducted in the years 1999–2001, which showed higher incidence of culture-positive typhoid fever in children aged 5–10 years^1^ and also a higher proportion of *S.* Typhi (85%) as compared with *S.* Paratyphi A (14%). The highest incidence of typhoid cases was seen in October (post-monsoon) followed by the months of May and June.^1^ A study conducted in children younger than 12 years in the years 1990–1993 showed 67.2% strains of *S.* Typhi to be multidrug-resistant (MDR),^14^ whereas multidrug resistance for *S.* Paratyphi A species was estimated at 44% in another study.^15^ A high prevalence of antimicrobial-resistant strains were found in a 3-year review (2008–2011) as *S.* Typhi was resistant to fluoroquinolone in 88.2% of the cases, followed by chloramphenicol, trimethoprim/sulfamethoxazole, and ampicillin at 66.8%, 66.5%, and 66.1%, respectively, and for *S.* Paratyphi A, fluoroquinolone resistance was highest at 83.9% followed by trimethoprim/sulfamethoxazole, chloramphenicol, and ampicillin at 83.9%, 2.6%, and 2.3%, respectively.^16^ Risk factors that contribute to the high prevalence of typhoid in this region include poor living conditions, overcrowding, poor sanitation, and lack of adequate water supply.^17^ A study conducted in the city of Karachi reported higher incidence of typhoid due to consumption of contaminated food and prior unnecessary antibiotic usage (as it alters the gut susceptibility to replication and invasion by *S.* Typhi).^18^ Safe drinking water is also associated with a 40% lower risk of typhoid.^19^ Vaccines are available in Pakistan; however, there are several factors which lead to its poor coverage, which includes lack of knowledge and awareness and limited ability to access immunization.^20,21^ Results from a vaccination trial conducted in Pakistan showed that the Vi polysaccharide vaccination was an effective strategy for the control of typhoid fever,^22^ whereas a more recent study suggested that the conjugate vaccine (Vi-CRM_197_) developed by the Novartis Vaccine for Global Health can be safely used in endemic population of all ages.^23^

## METHODS

### Data collection.

A retrospective cohort study on blood culture–positive typhoid fever was conducted from data of three large tertiary care hospitals in Pakistan: The Aga Khan University (AKU) in Karachi, Armed Forces Institute of Pathology (AFIP) in Rawalpindi, and Shaukat Khanum Memorial Cancer Hospital & Research Center (SKH) in Lahore. Two of these hospitals are situated in the province of Punjab, whereas one is situated in the province of Sindh. The Aga Khan University is a tertiary care hospital based in Karachi but has collection laboratories at various cities throughout Pakistan with the collection network growing over the years; SKH also has multiple collection laboratories across Pakistan, whereas AFIP is a major referral diagnostic center for military and civil hospitals. The Aga Khan University had five collection points in 1992 which increased to 224 points in 102 cities, whereas SKH had 26 collection points in 2004 which increased to 89 points in 38 cities. We obtained annual data from these three study hospitals; 1992–2015 for AKU, 2004–2015 for AFIP, and 2005–2014 for SKH. We also collected data on antibiotic resistance from these three institutes and the data were collected on all culture-positive specimens irrespective of age or gender.

The contextual factor data were obtained from the national household surveys conducted by national and international agencies. Data on water, sanitation and hygiene (WASH) indicators were obtained from the WHO/United Nations Children’s Fund Joint Monitoring Program database for water supply and sanitation^24^ and data on literacy, gross national income (GNI), and health expenditure were obtained from Pakistan Demographic and Health surveys (PDHS) and World Bank databases.^25^ The Joint Monitoring Program had data from various surveys conducted in different years (19 different survey years from 1991 to 2015), whereas PDHS was conducted in the year 1990–1991, 2006–2007, and 2012–2013. Data for missing values for any of the years were calculated using the FORECAST function of Microsoft Excel (Microsoft, Redmond, WA).

### Microbiological procedures and susceptibility testing.

Before the year 1997, each blood sample was collected in two blood culture bottles containing 45 mL of aerobic medium (brain heart infusion broth) and anaerobic medium (thioglycolate broth). The bottles were immediately transported to the laboratory and incubated at 37°C for 7 days. Gram stain film were made and bottles were subcultured after 24, 48, 72 hours, and after the seventh day of incubation on a blood agar and MacConkey’s media. Non–lactose-fermenting colonies from MacConkey’s plate were biochemically speciated using API 20E strips (bioMérieux, Marcy-l’Étoile, France). Serological confirmation was done by type-specific sera. From 1997 onward, blood was cultured using BACTEC system (Becton-Dickinson, Franklin Lakes, NJ) and for children younger than 5 years, BACTEC PEDS Plus bottles were used. On growth as indicated visually or by the BACTEC machine, the blood culture bottles were subcultured onto chocolate and MacConkey agar plates. The bottles were then incubated for 7 days before being discarded. Colonies giving biochemical reactions^26^ suggestive of *salmonellae* were confirmed serologically with specific O, Vi, and factor 9 antisera for *S.* Typhi, factor 2 antisera for Paratyphi A, and flagellar H(b) antisera for Paratyphi B (BD Laboratories, Franklin Lakes, NJ). *Salmonella* isolates were tested for antibiotic susceptibility by Kirby–Bauer disc diffusion method on Muller–Hinton agar with standard antimicrobial discs.^27^ *Salmonella* isolates were tested for ampicillin, ceftriaxone, chloramphenicol, co-trimoxazole, and fluoroquinolones in accordance with Clinical Laboratory Standards Institute (CLSI) guidelines.^28^

At AKU, during the study period (1992–2015), methodology to detect antimicrobial susceptibility to chloramphenicol, ampicillin, co-trimoxazole, and third-generation cephalosporins remained the same. However, for fluoroquinolones, the methodology was modified twice according to the recommendations of CLSI guidelines. Initially (1992–2000), antimicrobial susceptibility to ofloxacin/ciprofloxacin (5 mcg) and nalidixic acid (30 mcg) was checked by Kirby–Bauer disc diffusion method, nalidixic acid susceptibility was reported on a routine basis, but its resistance was not correlated with newer quinolones such as ofloxacin or ciprofloxacin. In the year 2000, interpretative criteria for fluoroquinolones were revised by CLSI.^29^ Therefore, from 2001 to 2011, the laboratory first performed disc diffusion test for all clinical isolates to screen nalidixic acid (30 μg) susceptibility, and isolates found resistant to nalidixic acid were further checked by performing ofloxacin minimum inhibitory concentration (MIC). A level of 0.125–1.0 μg/mL was used as reduced susceptibility and MIC of > 1.0 μg/mL as resistance to fluoroquinolones. In 2012, CLSI again changed its criteria of reporting; therefore, susceptibility of ofloxacin was reported only by disc diffusion test.^30^ It is important to note that because of this change in cutoffs, the number of intermediate resistance has increased and, thus, we report both the intermediate and resistant strains as resistant to fluoroquinolone.

### Data analysis.

We estimated positivity rates of *S.* Typhi/Paratyphi collectively and individually for the three hospitals using the number of blood culture–positive cases from the total number of blood cultures performed per year. *S.* Typhi/Paratyphi positivity rates and data on individual contextual factors were plotted by year to show the trend of each indicator over the years. We generated a locally weighted scatterplot smoothed for the yearly typhoid percent positivity for the three tertiary care hospitals. We then attempted to quantify the relative contribution of various contextual factors to reduce typhoid by exploring the correlation of typhoid and parathyroid positivity rates with the coverage of WASH indicators, literacy, and poverty rates, and economic growth (GNI per capita) at the national level through bivariate correlation analysis, and statistical analysis was performed using STATA.^31^

### Ethical approval.

Ethical approval was obtained from the Ethical Review Committee of AKU, AFIP, and SKH and the Research Ethics Board at SickKids, Toronto.

## RESULTS

We report data on a total of 17,387 *S.* Typhi (16,606 from AKU, 462 from SKH, 319 from AFIP) and 8,286 *S.* Paratyphi A and B (7,735 from AKU, 337 from SKH, and 214 from AFIP) blood culture–positive specimens from a total of 798,137 blood cultures performed at the three tertiary care hospitals and outreach laboratories in Pakistan and the largest dataset was from AKU.

### Positivity rates.

**Figure 1.**
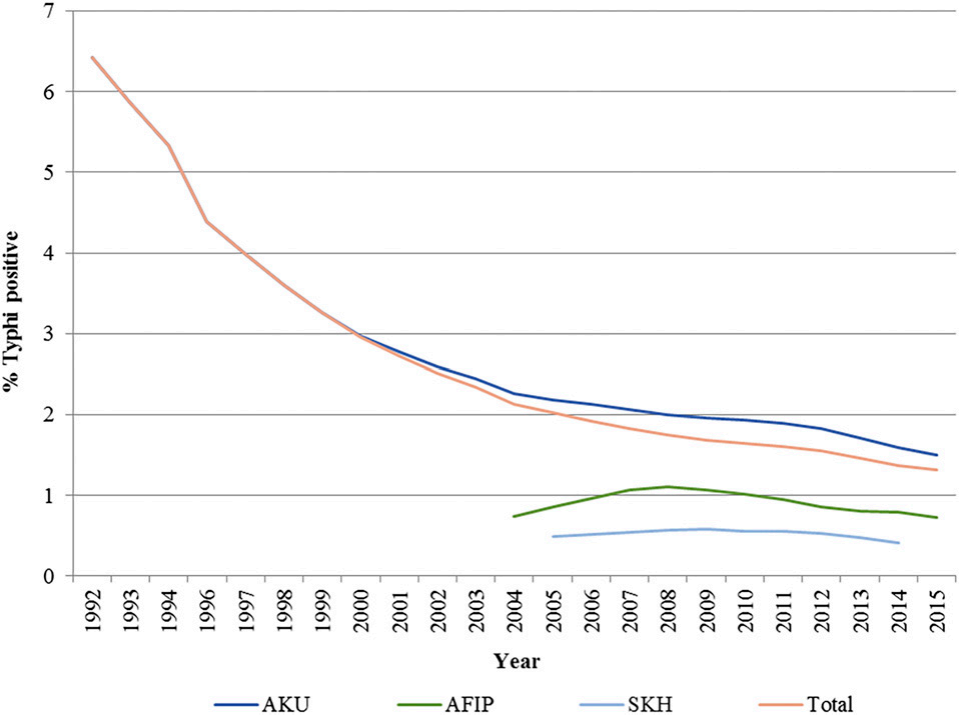
Positivity rates for *Salmonella* Typhi.

The results suggest that there has been an overall decline in the positivity rates of *S.* Typhi over the years, and the decline was marked from the year 1992 to 2004, but thereafter, there has been a slower reduction (Figure 1). Overall, the percentage contribution of *S.* Typhi to the total blood cultures performed has declined from 6.42% in 1992 to 1.32% in 2015. The disaggregated data from the three hospitals also suggest similar trends as shown in Figure 1, but the percentage contribution of *S.* Typhi to the total blood cultures is higher for AKU specimens than the other two institutes for all the years observed as shown in Figure 1. We performed a subgroup analysis by age which showed similar trends for children and adults. The positivity rates declined from 6.7% in 1992 to 2.2% in 2015 in children younger than 5 years, whereas the positivity rates declined from 18.6% to 5.2% for children aged 5–18 years during the same time period and for adults, it dropped from 34% to 5.7%.

**Figure 2.**
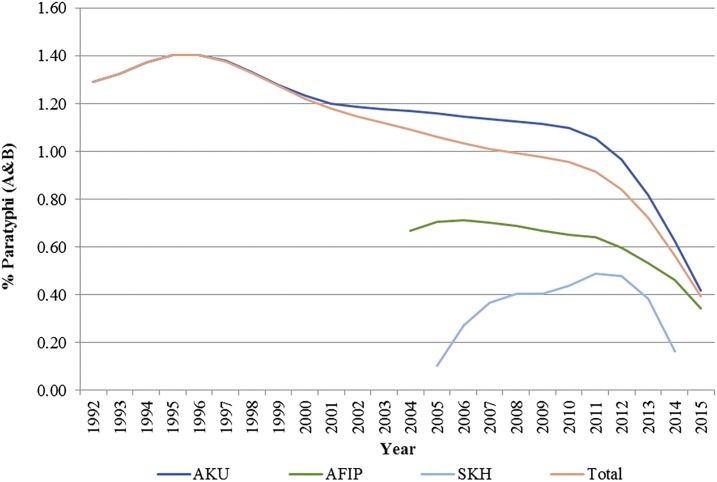
Positivity rates for *Salmonella* Paratyphi (A and B).

The trends for *S.* Paratyphi (A and B) also suggest decreased positivity rate from 1.29% in 1992 to 0.39% in 2015 (Figure 2). There has been a variable pattern with an initial rise, then a slow rate of decline, and a sudden dip from 2011 to 2012 onward, and this pattern is consistent for all the three hospitals. Subgroup analysis for age suggested a decline in positivity rates for *S.* Paratyphi from 0.7% to 0.23% in children younger than 5 years; 2.8% to 1.6% for children aged 5–18 years; and 1.2% to 0.37% in adults from 1992 to 2015. The relative contribution of *S.* Paratyphi to the overall typhoid-confirmed cases has also increased from 16.8% in 1992 to 23% in 2015, suggesting a greater decline for *S.* Typhi as compared with *S.* Paratyphi.

### Antimicrobial resistance.

We report data on AMR from the three institutes and classified the strains as MDR (resistant to ampicillin, chloramphenicol, and co-trimoxazole) and fluoroquinolone (ciprofloxacin and/or ofloxacin) resistant.

**Figure 3.**
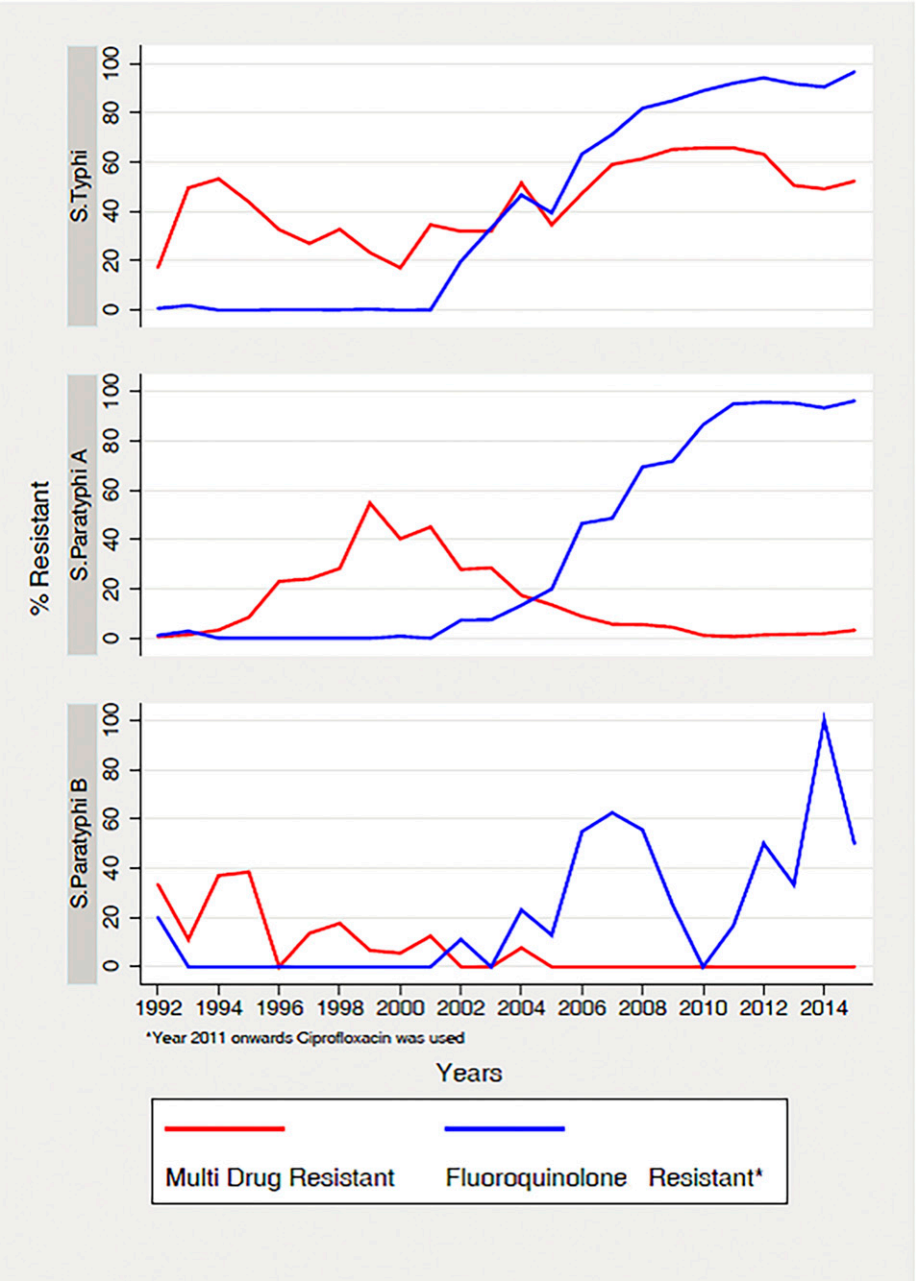
Data for antimicrobial resistance patterns from 1992 to 2015 at The Aga Khan University.

Figure 3 shows the trends for *S.* Typhi and *S.* Paratyphi AMR at AKU which has the largest sample and duration from 1992 to 2015. The data suggests high prevalence of MDR strains of *S.* Typhi, rising from around 20% in 1992 to around 50% in 2015, whereas the MDR *S.* Paratyphi A and B strains have declined since the year 2004 to almost negligible levels. Fluoroquinolone resistance for *S.* Typhi started rising in the year 2002 and reached to 96.5% in 2015, and similar patterns were observed for *S.* Paratyphi A with a resistance of 96.2% in the year 2015. Data from AFIP and SKH also suggest high prevalence of multidrug resistance and fluoroquinolone resistance for both *S.* Typhi and *S.* Paratyphi.

### Contextual factor trends and associations.

**Figure 4.**
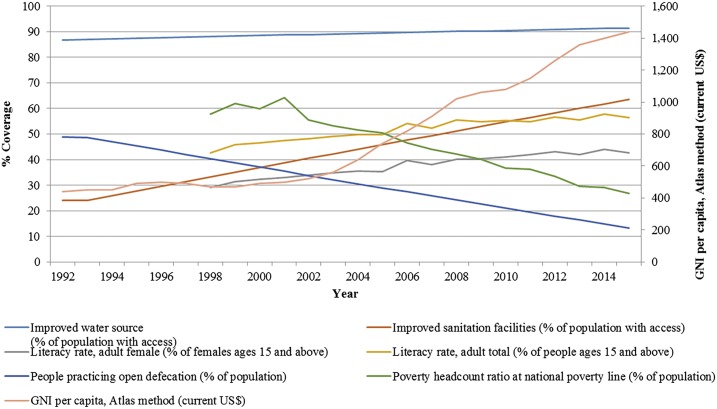
Trends in contextual factors in Pakistan from 1992 to 2015.

Figure 4 depicts the trends in various contextual factors in Pakistan, including WASH indicators (water supply, sanitation, and open defecation), literacy, and poverty rates and GNI per capita from 1992 to 2015. Overall, there has been a positive trend in all the contextual factors. Coverage of improved water supply has increased from 86% in 1992 to 91% in 2015, improved sanitation from 24% to 64%, whereas open defecation rates have reduced from 49% to 13%. Literacy rates have improved from 42% in 1998 to 56% in 2015, whereas female literacy rates have improved from 29% to 43% during the same time period. Poverty headcount ratio has decreased from 58% in 1998 to 30% in 2013, whereas there has been a gradual increase in GNI per capita to USD 1,440 in 2015.

Table 1 suggests a statistically significant association of all the contextual factors with *S.* Typhi positivity rates, with the WASH indicators and literacy levels suggesting a strong association, whereas the association of *S.* Paratyphi is significant for WASH indicators.

**Table 1:** Table showing association of typhoid with various contextual factors: correlation coefficient (*P* value)

	Improved water source (percentage of population with access)	Improved sanitation facilities (percentage of population with access)	People practicing open defecation (percentage of population)	Literacy rate, adult female (percentage of females ages 15 and above)	Literacy rate, adult total (percentage of people ages 15 and above)	Poverty headcount ratio at national poverty line (percentage of population)	Gross national income per capita, Atlas method (current US$)	Health expenditure, total (percentage of GDP)
*Salmonella* Typhi positivity rate	−0.87 (0.0000)	−0.85 (0.0000)	0.85 (0.0000)	−0.81 (0.0049)	−0.82 (0.0033)	0.79 (0.021)	−0.68 (0.0003)	−0.45 (0.0454)
*Salmonella* Paratyphi	−0.52 (0.009)	−0.53 (0.0081)	0.53 (0.008)	−0.50 (0.1399)	−0.50 (0.1455)	0.47 (0.2425)	−0.49 (0.0143)	0.09 (0.6959)

GDP = gross domestic product.

## DISCUSSION

The study suggests that there has been a decline in the cases of typhoid fever in Pakistan since 1992. *Salmonella* Typhi is more prevalent in adults, whereas *S.* Paratyphi is more prevalent in children aged 5–18 years, although all age groups have observed significant decline over the years. There was greater decline for *S.* Typhi from 1990 to 2004, although the rate of decline has reduced, whereas for *S.* Paratyphi, initially there was a marginal decline but after 2011, a rapid decline was observed. These trends and age patterns are consistent with other studies conducted in the country.^1,13^ We also looked at the data from the city of Hyderabad separately, owing to the recent outbreak of ceftriaxone-resistant strain,^32^ and the trends from the year 1991 suggests a steady decline in *S.* Typhi from 4% to 2.2% in the year 2015 and *S.* Paratyphi A from 3.4% to 0.4% during the same time period, and these are similar to the national trends.

The analysis suggests a statistically strong association of WASH indicators and literacy levels with typhoid. Although this is not a robust analysis, evidence from this and other studies in similar contexts is comprehensive enough to suggest that typhoid control would require improvements in contextual factors, including female literacy, sanitation, and hygiene conditions.^33–35^ There was no reliable data for food safety in Pakistan, but the importance on focusing on improving food safety protocols and implementation cannot be undermined.^34,36^ The results of our study also suggest a high burden of MDR and fluoroquinolone-resistant strains, and this could partly be attributed to the routine practice of judicious antibiotic use in Pakistan, especially by general practitioners, and easy availability of antibiotics.^37^

The data presented are from three large tertiary care hospitals in the region and two of the institutes have a wide network of laboratory specimen collection points spread across the country; thus, our sample includes inpatients and patients from the community across Pakistan and the data are present for the last 25 years; hence, the results are reflective for the trends of typhoid in Pakistan. The limitation of our study is that it is based on laboratory data and cannot correctly calculate the existing prevalence, whereas information of disease severity, complications, and mortality are also missing. There are currently no national or subnational public data repositories for typhoid surveillance in Pakistan, highlighting the need to set up surveillance systems with proper recordkeeping and reporting mechanisms in health-care facilities nationally. An expert consensus should also be established on the recommended diagnostic criteria, and quality-assured laboratories should be made available at facilities with standardized diagnostic methods.

A multipronged approach is required specifically focusing on improved WASH and food safety which are related to improvements in literacy and economic independence. These factors are a prerequisite if a long-term sustainable solution is to be achieved. Large-scale immunization could be a short-to-medium term measure in the endemic settings of Pakistan and to prevent epidemics, and this is of even more relevance considering the recent emergence of ceftriaxone-resistant strains.

Control on typhoid can only be actualized if adequate surveillance systems are put in place to constantly monitor the longitudinal trends on typhoid fever and evaluate AMR and its public health consequences. A mass awareness campaign is desired, focusing on public and private practitioners for appropriate use of diagnostic tools and antibiotics for typhoid, and focusing on preventive strategies cannot be overemphasized. Countries with similar contexts have made greater strides in reducing the typhoid burden, and Pakistan should step up to reduce the incidence of typhoid and typhoid-related morbidity and mortality.
